# StepFit-18K: Improving Irritable Bowel Syndrome (IBS) Symptoms with a Simple, Structured Walking Intervention

**DOI:** 10.3390/jcm13226684

**Published:** 2024-11-07

**Authors:** Isabella Franco, Antonella Bianco, Laura Prospero, Giuseppe Riezzo, Caterina Bonfiglio, Claudia Beatrice Bagnato, Nicola Verrelli, Francesco Goscilo, Benedetta D’Attoma, Antonia Ignazzi, Sergio Coletta, Maria Grazia Refolo, Rossella Donghia, Francesco Russo

**Affiliations:** 1 Laboratory of Movement and Wellness, National Institute of Gastroenterology IRCCS “Saverio de Bellis”, 70013 Castellana Grotte, Italy; isabella.franco@irccsdebellis.it (I.F.); antonella.bianco@irccsdebellis.it (A.B.); claudia.bagnato@irccsdebellis.it (C.B.B.); nicola.verrelli@irccsdebellis.it (N.V.); 2 Functional Gastrointestinal Disorders Research Group, National Institute of Gastroenterology IRCCS “Saverio de Bellis”, 70013 Castellana Grotte, Italy; laura.prospero@irccsdebellis.it (L.P.); giuseppe.riezzo@irccsdebellis.it (G.R.); francesco.goscilo@irccsdebellis.it (F.G.); benedetta.dattoma@irccsdebellis.it (B.D.); antonia.ignazzi@irccsdebellis.it (A.I.); 3 Data Science Unit, National Institute of Gastroenterology IRCCS “Saverio de Bellis”, 70013 Castellana Grotte, Italy; catia.bonfiglio@irccsdebellis.it (C.B.); rossella.donghia@irccsdebellis.it (R.D.); 4 Core Facility Biobank, National Institute of Gastroenterology IRCCS “Saverio de Bellis”, 70013 Castellana Grotte, Italy; sergio.coletta@irccsdebellis.it; 5 Laboratory of Clinical Pathology, National Institute of Gastroenterology IRCCS “Saverio de Bellis”, 70013 Castellana Grotte, Italy; maria.refolo@irccsdebellis.it

**Keywords:** gastrointestinal symptom, health promotion, irritable bowel syndrome, physical activity, walking

## Abstract

**Background:** Irritable Bowel Syndrome (IBS) is a chronic functional gastrointestinal (GI) disorder characterized by abdominal pain, altered bowel habits, and bloating, affecting approximately 10.1% of the global population. While current treatments emphasize dietary modifications and symptom management, emerging evidence suggests that physical activity (PA) may help alleviate IBS symptoms. This study evaluated the effects of a structured walking program, “StepFit-18K”, on IBS symptom relief. **Methods:** A total of 106 participants (68 females and 38 males) completed the 12-week intervention, which consisted of 18,000 additional steps per week (6000 extra steps on three days per week) tracked via fitness devices. The program emphasized step count, ease of adoption, and social support through walking groups. **Results:** As assessed by validated questionnaires, significant reductions in GI symptoms were observed. The IBS Symptom Severity Score (IBS-SSS) decreased from 118.30 ± 98.73 to 74.46 ± 74.93, with a delta score of −43.84%, highlighting bloating as the most improved symptom. No clinically significant changes were observed in anthropometric or biochemical markers. **Conclusions:** StepFit-18K is a simple, accessible, and effective form of physical activity that has demonstrated therapeutic benefits for IBS symptoms. This offers an additional application beyond its well-established role in preventing cardiovascular diseases.

## 1. Introduction

Irritable Bowel Syndrome (IBS) is a chronic functional bowel disorder that can significantly debilitate patients and is characterized by recurrent episodes of abdominal pain, altered bowel habits, bloating, and flatulence [1].

Accurately determining a single global prevalence rate for IBS is difficult due to significant variations in study designs and population samples. Recent surveys estimate the prevalence at around 10.1% [2], with young adults, especially women, being the most affected [3]. This condition has been found to decrease the quality of life (QoL) and work habits, increasing direct and indirect healthcare costs [3]. The exact etiological causes of IBS remain unclear. Still, due to its complex nature and wide range of symptoms, research focuses on identifying its underlying mechanisms, including the effects of diet, genetics, and environmental factors [4].

The above-cited risk factors are not absolute, as several modifiable ones, such as smoking, sleep, diet, physical activity (PA), and alcohol, have been independently associated with IBS [5].

The current management methods for IBS include symptom control, the improvement of bowel functions, and pain reduction as primary goals of therapy supplemented by dietary modifications. Randomized clinical trials have shown that changing dietary habits, removing specific foods or adhering to the low Fermentable Oligosaccharides Disaccharides Monosaccharides and Polyols (low-FODMAPs) exclusion diet is more effective than a regular diet in ameliorating the signs, symptoms, inflammation, and gut permeability of IBS patients [6,7,8,9].

Beyond diet, PA may be an effective treatment for chronic conditions, such as functional gastrointestinal (GI) disorders like IBS [10,11]. Incorporating PA into daily routines helps improve health and lessen IBS symptoms, fostering empowerment and hope.

Previous studies have highlighted PA’s positive effects on the GI system, alleviating symptoms like gas retention, abdominal distension, and bowel discomfort [12]. Biologically, PA increases intestinal motility, enhances mucosal immunity, and has effects beyond the pelvic cavity [13]. PA is presumed to reduce the risk of GI disorders, manage IBS symptoms, and improve psychological well-being [14]. Research on 6 to 24 weeks of supervised PA interventions shows significant reductions in GI symptoms compared to standard therapy [2,15].

Guidelines recommend that IBS patients engage in 30 to 60 min of moderate-intensity aerobic exercise, like walking, 3 to 5 days a week [16]. Walking is popular due to its low injury risk, no need for equipment, and flexibility in any setting [17].

Systematic reviews have provided evidence of the significant health benefits of regular walking, especially at 5–8 km/h. Therefore, walking is internationally recommended for physically active people with IBS [18]. In addition, outdoor walking, or “Green Exercise”, which involves exercising in green spaces, often in groups, is also promoted to improve PA levels and mental health for patients with psychosomatic illnesses [19].

In our experience, a walking group is a practical therapeutic support due to its motivational and social characteristics. It promotes high participation, encourages local engagement, is enjoyable, and provides safety and supervision by qualified personnel [20].

Nevertheless, many studies agree that the number of daily steps, rather than their intensity, plays a key role in maintaining good health [21,22]. Available data have indicated that healthy adults generally take between 4000 and 18,000 steps per day and that the threshold of 10,000 steps per day could be a sufficient indicative value [23].

In a report by the National Health Promotion Movement in 21st Century Japan (Health Japan 21), recommendations on daily PA to keep healthy are set at 9000 steps for males and 8500 for females aged 20 to 64 [24]. Cadence and step intensity, particularly in studies on walking, have shown a positive correlation, with 100 steps per minute suggested as a baseline standard for moderate-intensity walking [23].

Additionally, affordable PA fitness trackers have transformed how PA is monitored due to their low cost and the exact and instantaneous assessments of daily steps and other variables. These devices enhance motivation through features like goal setting, rewards, and social sharing, which encourage consistent PA. Increasing awareness and providing continuous feedback encourage users to make healthier daily choices and maintain dedication and accountability to their fitness objectives. These trackers also offer personalized insights and plans integrated with mobile apps, making them powerful tools for improving PA and overall health [25].

In this framework, we aimed to examine the effect of increased PA in the form of walking on IBS symptoms. This study aimed to determine whether IBS patients participating in a PA program based on walking 18,000 steps per week (6000 steps per day, three days per week), monitored by fitness trackers and professional supervision (StepFit-18K), would experience a significant improvement in their IBS symptoms.

## 2. Materials and Methods

### 2.1. Participants

The project participants were recruited by the Functional Gastrointestinal Disorders Research Group in collaboration with the Laboratory of Movement and Wellness of the National Institute of Gastroenterology IRCCS “Saverio de Bellis” in Castellana Grotte, Italy. Recruitment began in May 2022 and ended in December 2023. The study was registered at https://clinicaltrials.gov/ (last accessed on 8 July 2022) (registration number NCT05453084). 

### 2.2. Study Design

This study enrolled subjects with IBS (Rome III–IV criteria) referred to the clinic for celiac disease and functional disorders or by general practitioners of Castellana Grotte.

The exclusion criteria were as follows: (1) severe neurological, hepatic, psychiatric, and cardiac pathologies; (2) having previously followed a low FODMAPs, vegan, or gluten-free diet; (3) GI disorders other than IBS (i.e., Inflammatory Bowel Disease and metabolic syndrome); (4) pharmacological treatment with antidepressants; (5) musculoskeletal disorders that may prevent regular physical exercise; (6) absolute contraindications to physical exercise; (7) pregnancy; and (8) cancer, chemotherapy, or immunotherapy.

The inclusion criteria were as follows: (1) age (18–65 years); (2) willingness to take part in group walks; and (3) suitability for non-competitive sports. This study was conducted according to the Declaration of Helsinki and was approved by the local Ethics Committee (Prot. N. 167/CE 2022 De Bellis).

### 2.3. Data Collection

All study participants signed an informed consent form and answered questions about their general psychophysical state before formally enrolling in the research.

All subjects completed two validated GI questionnaires—the Gastrointestinal Symptom Rating Scale (GSRS) [26], which uses a 7-level Likert scale (merged into 4 levels: absent, mild, moderate, and severe, as published previously [27]) to assess symptom intensity and frequency over the past seven days, and the IBS Symptom Severity Score (IBS-SSS) [28], which measures the severity of IBS symptoms (pain, bloating, bowel habits, and impact on daily life) with a total score ranging from 0 to 500. The International Physical Activity Questionnaire—Short Form (IPAQ-SF) [29] assessed PA levels, categorizing them as low, moderate, or high based on activity type and duration in the previous week.

**Figure 1:**
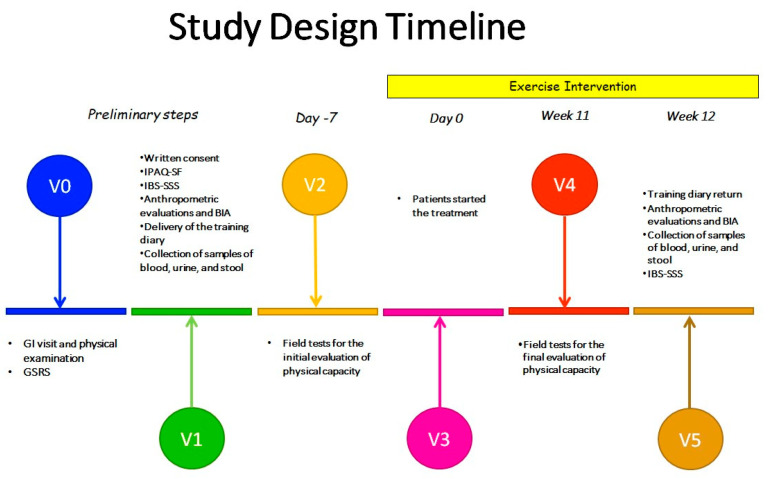
Study timeline. GI: gastrointestinal; GSRS: Gastrointestinal Symptom Rating Scale; IPAQ-SF: International Physical Activity Questionnaire—Short Form; IBS-SSS: Irritable Bowel Syndrome -Severity Scoring System; BIA: bioimpedance analysis; VO: preliminary screening; V1: initial visit and general evaluation; V2: field test initial evaluation; V3: start of the treatment; V4: field test final evaluation; V5: final evaluation.

Specialized nurses collected blood and stool samples, while dieticians performed bioimpedance analysis (BIA) and collected anthropometric measurements (body mass index (BMI), as well as waist, hip, arm, calf, wrist, and neck circumferences). The BIA 101 BIVA PRO instrument (Akern SRL, Pontassieve, Italy) was used to perform BIA, and the collected data were calculated using specialized software (Bodygram PLUS Software v. 1.0, Akern SRL, Pontassieve, Italy). All patients were informed about the procedure to be followed to prepare for the BIA [30]. The timing of the study design is illustrated in Figure 1.

Each participant was given a step diary to manually fill in after adequate explanations to monitor and quantify the daily PA. For correct compilation, an example table and a legend were included. All subjects had to report the duration and type of PA performed outside the project and when they had walked in a group at the end of the day. The training diary not only served as a motivational tool, but also enabled participants to compare their attendance with the records kept by the kinesiologists.

### 2.4. Clinical Biochemistry

Blood samples were collected in the day between 8 a.m. and 9 a.m. after an overnight fast.

The COBAS 8000 autoanalyzer (ROCHE Diagnostic SPA, Monza, Italy) was used to measure levels of fasting blood glucose, insulin, triglyceride, total cholesterol, low-density lipoprotein cholesterol (LDL-cholesterol), high-density lipoprotein cholesterol (HDL-cholesterol), total and direct bilirubin, creatinine, estimated glomerular filtration rate (eGFR), urea, iron, ferritin, aspartate transaminase (AST), alanine transaminase (ALT), gamma glutamyl transpeptidase (GGT), vitamin B12, calcium, intact parathormone, and 25-OH vitamin D. The automatic system for capillary electrophoresis—Capillarys 3 OCTA (Sebia Italia S.r.l., Bagno a Ripoli, Firenze, Italy)—was used to determine the levels of glycosylated hemoglobin (HbA1c).

Thyrotropin (TSH), triiodothyronine (FT3), and tetraiodothyronine (FT4) serum concentrations were measured using a competitive luminometric assay based on the SPALT (solid-phase antigen luminescence technique) principle (LIAISON FT3, FT4, TSH, DiaSorin, Saluggia, Italy).

### 2.5. Exercise Program

#### 2.5.1. Fitness Assessment: “Field Test”

In our study, three “Field Tests” were used to assess cardiorespiratory capacity (2Walking Test) [31], strength (Hand Grip Test) [32], and the flexibility of the posterior chain (Sit and Reach Test) [33]. All participants were informed about the purpose of the tests and how they were performed. Furthermore, during walks the previous week, kinesiologists trained the subjects to use the heart rate monitor properly. They taught them the correct walking technique, paying particular attention to using the arms, legs, and breathing to prepare them adequately. The American College of Sports Medicine’s table was used to evaluate the results of the three standardized tests, which were administered at the same time and location and with the same measuring equipment and explanations [34].

#### 2.5.2. Study Protocol

Fitwalking [35], a moderate-intensity aerobic activity, was used as an intervention tool. The walk intensity was standardized between 60 and 75% of the maximum heart rate predicted by age [36], monitored by a heart rate monitor, the Talk Test [37], and the perception of fatigue through the Borg scale [38]. Kinesiologists instructed the participants on using the smartwatch to monitor heart rate in real time. The walks were carried out in the urban and extra-urban areas of Castellana Grotte. The intervention lasted 12 weeks, during which the walking group met thrice a week on non-consecutive days for 60 min. All walks were carried out with the walking group, which was organized by our research hospital and conducted under the supervision of qualified personnel. The outings took place in all weather conditions except in the case of rain. Attendance was recorded each time the activity was carried out.

**Figure 2:**
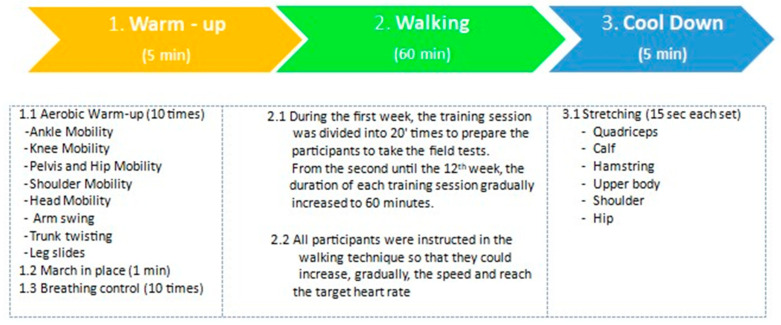
The StepFit-18K intervention.

The intervention (called StepFit-18K) was standardized on 6000 steps per 60′ of walking (18,000 per week for 12 weeks), considering that 100 steps/per minute is an indicative value of moderate aerobic activity [23] (Figure 2).

### 2.6. Statistical Analysis

Subject characteristics are reported as mean and standard deviation (M ± SD) for continuous variables, and as frequency and percentages (%) for categorical variables. The Wilcoxon matched-pairs signed-rank test for continuous variables was applied to evaluate pre/post-PA variations. For categorical variables, the McNemar or McNemar–Bowker test was used where necessary. To test the null hypothesis of no association, a two-tailed significance level was set at 0.05. The analyses were conducted with StataCorp 2023 (Stata Statistical Software: Release 18, College Station, TX, USA, StataCorp LLC), while RStudio (“Chocolate Cosmos” Release) was used for the plot. The exposure variable was the standardized number of steps per hour of moderate-intensity walking [23].

## 3. Results

**Figure 3:**
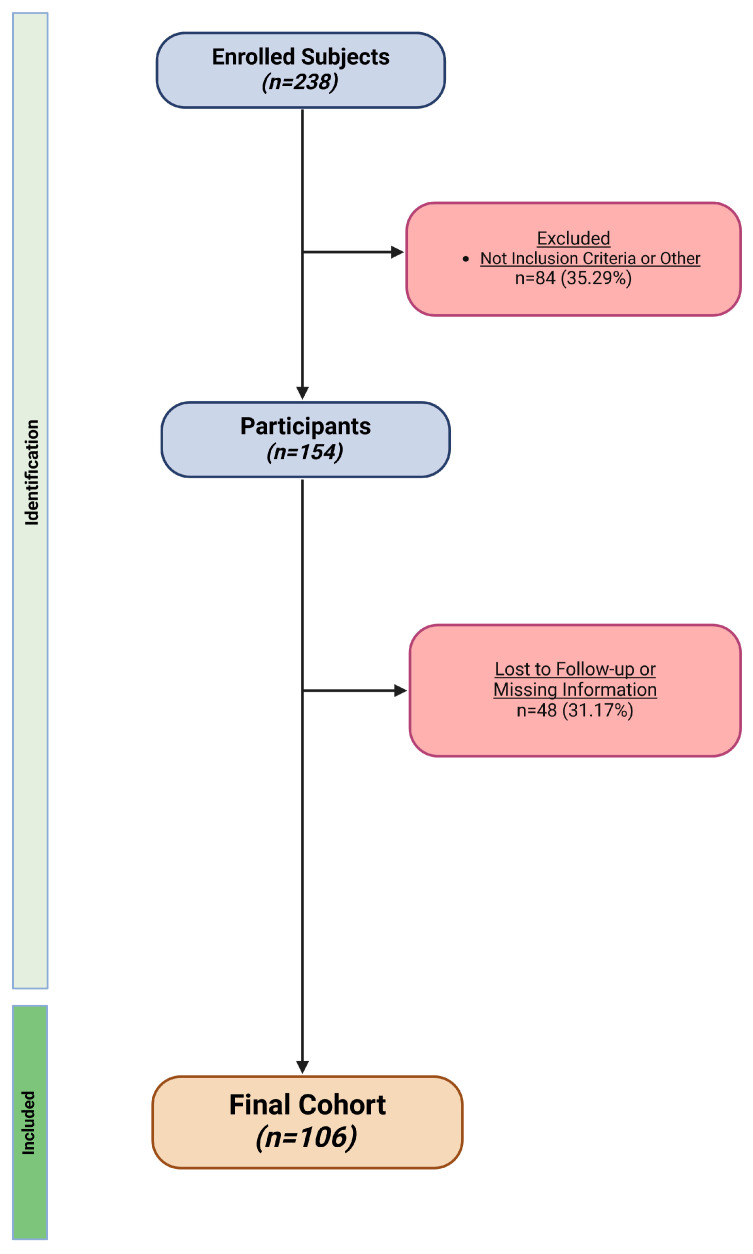
The flowchart of the study. Image created with BioRender (accessed on 04 September 2024).

Out of the 238 subjects initially recruited, 84 (35.29%) were excluded because they did not meet the minimum required criteria. Among the remaining 154 enrolled participants, 48 (31.17%) did not complete the follow-up for different reasons (work or health problems, moved to another city, and/or missing information about any parameters). Ultimately, data from 106 participants were analyzed (Figure 3).

The group’s composition was predominantly female, with 64.15% of the participants being women, compared to 35.85% being men (F = 68 and M = 38). The average age of the participants was 52.32 ± 7.95 years

Notably, 94% of participants completed over 60% of the total project activities, firmly adhering to the StepFit-18K program.

Table 1 presents the statistically significant changes in anthropometric parameters after the StepFit-18K intervention, expressed as BMI (ΔPost-Pre −0.33; *p* = 0.004), waist circumference (Δ_After-Before_ −1.11; *p* = 0.009), hip circumference (Δ_After-Before_ −0.83; *p* = 0.0004), arm circumference (Δ_After-Before_ −0.43; *p* = 0.001), wrist circumference (Δ_After-Before_ −0.08; *p* = 0.004), and neck circumference (Δ_After-Before_ −0.32; *p* = 0.002). No statistically significant changes were observed in calf circumference (Δ_After-Before_ +0.15; *p* = 0.10).

Additionally, BIA parameters showed borderline significant differences in extracellular water (Δ_After-Before_ −0.11; *p* = 0.06) and statistically significant in fat mass (Δ_After-Before_ −0.77; *p* = 0.01). All other parameters did not demonstrate substantial differences.

As reported in Table 2, there was a decrease in glycosylated hemoglobin (Δ_After-Before_ −1.02; *p* = 0.0003) and intact parathormone (Δ_After-Before_ −11.92; *p* = 0.0004). Conversely, calcium (Δ_After-Before_ +0.16; *p* < 0.0001) and triglyceride levels (Δ_After-Before_ +9.47; *p* = 0.01) rose significantly, though they remained within the normal range, suggesting that the intervention did not negatively affect lipid metabolism. All the other parameters did not show significant changes.

**Figure 4:**
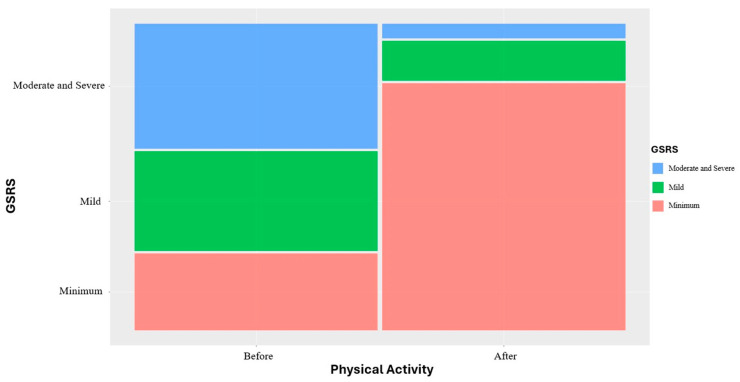
Mosaic plot of GSRS categories before and after StepFit-18K.

Before the intervention, 27 subjects (24.47%) had minimal GSRS scores, 35 (33.02%) had mild symptoms, and 44 (41.51%) had moderate to severe symptoms. After the intervention, 87 subjects (82.08%) reported minimal GSRS scores, 14 (13.21%) had mild symptoms, and only 5 (4.72%) remained in the moderate to severe category. This finding demonstrates a statistically significant improvement (*p* < 0.0001), as shown in Figure 4.

Table 3 shows the improvement in IBS-SSS parameters following PA intervention. Notably, there is a statistically significant reduction in abdominal pain intensity (Δ_After-Before_ −9.06; *p* < 0.0001), abdominal pain frequency (Δ_After-Before_ −7.27; *p* < 0.0001), abdominal distension severity (Δ_After-Before_ −11.40; *p* < 0.0001), and dissatisfaction with bowel habits (Δ_After-Before_ −8.66; *p* = 0.004), as well as a decrease in urgency (Δ_After-Before_ −16.65%; *p* = 0.0002) and the sensation of incomplete evacuation (Δ_After-Before_ −18.65%; *p* = 0.0003). Also, interference with life in general (Δ_After-Before_ −7.46, *p* = 0.006) and IBS-SSS Total Score (Δ_After-Before_ −43.84 *p* < 0.0001) had a strong and statistically significant reduction after StepFit-18K. Abdominal bloating, rather than abdominal pain, is confirmed as the predominant symptom, accompanied by dissatisfaction with bowel habits.

## 4. Discussion

Scientific evidence consistently proves that regular PA offers many health benefits [39] and helps prevent major non-transmissible chronic diseases [40]. As a result, PA has become a very successful therapeutic option, particularly in managing conditions such as IBS, where pharmacological treatments often result in limited or inconsistent relief [41]. Our study demonstrated how a simple and cost-effective program like StepFit-18K can have therapeutic effects on IBS symptoms. This represents an additional application beyond its recognized efficacy in cardiovascular disease prevention and its growing validity in managing functional gastrointestinal disorders. We included IBS patients without metabolic syndrome to isolate its impact on GI symptoms. As a result, no clinically significant changes were observed in biochemical or anthropometric variables among the participants. Our study adds to the growing evidence suggesting that IBS patients can significantly improve their overall health and reduce their GI symptoms when applying even a moderate amount of PA [20].

Specifically, this walking-based aerobic exercise program was developed for three months, during which participants increased their daily activity by 6000 steps a day, three days a week. This structured approach gave participants a clear and tangible goal, offering a simple yet effective means to increase daily PA levels. The World Health Organization recommends taking at least 10,000 steps daily to maintain good health and prevent inactivity [21]. According to our study, increasing weekly steps by 18,000 can reduce the symptom score. This applies to the total score and individual items, including abdominal pain intensity and frequency, abdominal bloating severity, dissatisfaction with bowel habits, and interference with life in general. Notably, bloating, rather than abdominal pain, emerged as the predominant symptom in patients with IBS.

Overall, our intervention had no clinically relevant impact on most physiological markers, including glycated hemoglobin, which other studies have shown to significantly decrease after physical activity, with notable improvements in glucose metabolism [42]. Other parameters, such as intact parathyroid hormone, calcium, and triglyceride levels, although showing statistically significant changes, remained within the normal range and had no clinical relevance, indicating the absence of adverse metabolic effects in a population like ours without metabolic alterations.

The link between step counts and health outcomes is well documented. Studies show that increased step counts correlate with reductions in BMI, body fat percentage, and waist circumference, all of which contribute to improved health [43,44].

Since the 1990s, step counting has evolved from merely measuring walking distance to becoming a comprehensive PA indicator. It is now recognized as a reliable and straightforward measure of overall PA, with higher step counts consistently associated with improved health outcomes [45,46].

StepFit-18K may be easily integrated into daily schedules, is accessible to many people, and does not require specialized technology or advanced preparation. Its simplicity makes it an extremely effective persuasive approach for improving PA levels in people with IBS.

Fitness trackers were essential to our participants’ ability to monitor their progress in real time. These devices recorded step counts and provided continuous feedback through apps and wearable displays, allowing participants to visualize their achievements and stay motivated. These devices’ real-time feedback enhances accountability, as participants can track their adherence to prescribed activity levels. Moreover, fitness trackers are affordable, easy to use, and widely available, making them an ideal tool for clinical settings and personal use [47,48].

An innovative aspect of our program was the outdoor walking group, which added a valuable social dimension. Group participation fostered socialization, peer support, and motivation, improving adherence. These psychological benefits, especially for IBS patients who often face isolation due to unpredictable symptoms, were integral to the program’s success [49,50]. Group dynamics made PA more enjoyable, reducing perceived exertion and promoting long-term sustainability [51,52].

Outdoor walking offered additional mental health advantages, such as reduced stress and improved mood, through exposure to nature. These benefits are particularly relevant for IBS patients, whose symptoms are often exacerbated by stress [53].

The standardized, step-based structure of StepFit-18K made it easy for physicians to prescribe and participants to follow, with clear, achievable goals. The combination of group enjoyment, expert supervision, and self-monitoring with fitness trackers contributed to the high participation rates observed in this study.

Despite the clear strengths of our study, some limitations must be acknowledged. The relatively small sample size may have limited the statistical power of our results, and the 12-week intervention period, while sufficient to observe significant changes, may not have been long enough to evaluate the long-term effects of PA on IBS symptoms fully. Additionally, the lack of an active control group limited our ability to isolate the specific impact of walking from other forms of exercise or non-physical interventions. Finally, while participants used fitness trackers to monitor their steps, self-monitoring may have introduced some degree of bias into the measurements.

## 5. Conclusions

This study provides evidence for the effectiveness of a structured walking program based on a fixed step count as a non-pharmacological intervention for managing IBS. By integrating PA into daily life by adding 18,000 weekly steps, patients can experience significant improvements in GI symptoms and overall QoL.

The simplicity, accessibility, and motivational aspects of this approach, enhanced by real-time feedback from fitness trackers and the social support provided by the walking group, make it an appealing option for both clinicians and patients. In this context, step-count-based walking programs could serve as a valuable complement to standard treatment protocols for this debilitating condition.

Future studies should explore the long-term effects of step-count-based PA on IBS symptoms, QoL, and variations in PA dosage to optimize symptom relief. Including IBS subtypes and patients with different comorbidities could further personalize interventions. Additionally, digital health tools and remote group dynamics may enhance accessibility and adherence, broadening the impact of programs like StepFit-18K for IBS management

## Figures and Tables

**Table 1:** Variation in anthropometric BIA parameters before and after the StepFit-18K intervention.

Parameters *	StepFit-18K		Δ_After-Before_	*p* ^^^
	Before	After		
BMI (kg/m^2^)	28.79 ± 0.50	28.46 ± 0.50	−0.33	**0.004**
Waist (cm)	93.43 ± 1.32	92.32 ± 1.30	−1.11	**0.009**
Hips (cm)	105.30 ± 0.95	104.46 ± 0.93	−0.83	**0.0004**
Arm (cm)	33.35 ± 0.39	32.92 ± 0.40	−0.43	**0.001**
Calf (cm)	37.71 ± 0.38	37.86 ± 0.36	+0.15	0.10
Wrist (cm)	17.20 ± 0.14	17.13 ± 0.14	−0.08	**0.004**
Neck (cm)	37.71 ± 0.39	37.39 ± 0.38	−0.32	**0.002**
ECW	16.53 ± 0.29	16.41 ± 0.31	−0.11	0.06
FM	25.85 ± 1.04	25.08 ± 1.07	−0.77	**0.01**

StepFit-18K was standardized on 6000 steps per 60′ of walking for three days per week for three months. * Data reported as mean and standard deviation for continuous variables. ^ Wilcoxon matched-pairs signed-rank test. Abbreviations—BMI: body mass index; ECW: extracellular water; FM: fat mass.

**Table 2:** Variation in blood parameters before and after the StepFit-18K intervention.

Parameters *	StepFit-18K		Δ_After-Before_	*p* ^^^
	Before	After		
Glucose (mg/dL)	93.37 ± 1.97	92.43 ± 1.27	−0.93	0.84
HbA1c (mmol/mol)	36.16 ± 0.62	35.14 ± 0.35	−1.02	**0.0003**
Urea (mg/dL)	37.70 ± 0.86	38.18 ± 0.98	+0.48	0.69
Creatinine (mg/dL)	0.83 ± 0.014	0.83 ± 0.01	+0.002	0.99
eGFR (mL/min)	80.71 ± 0.87	80.07 ± 0.93	−0.64	0.35
DB (mg/dL)	0.19 ± 0.01	0.19 ± 0.07	+0.01	0.84
TB (mg/dL)	0.62 ± 0.03	1.27 ± 0.59	+0.65	0.06
AST (U/L)	21.85 ± 0.63	22.93 ± 1.74	+1.08	0.06
ALT (U/L)	24.60 ± 1.23	26.41 ± 2.31	+1.81	0.31
GGT (U/L)	24.61 ± 2.30	25.83 ± 2.88	+0.76	0.92
Calcium (mg/dL)	4.56 ± 0.02	4.72 ± 0.27	+0.16	**<0.0001**
Iron (μg/dL)	92.20 ± 3.24	91.55 ± 3.03	−0.64	0.92
Ferritin (ng/mL)	141.72 ± 13.56	135.49 ± 13.37	−6.23	0.06
TC (mg/dL)	202.29 ± 3.43	202.23 ± 3.56	−0.06	0.55
HDL (mg/dL)	58.56 ± 1.65	57.39 ± 1.76	−1.18	0.09
LDL (mg/dL)	125.81 ± 3.13	127.55 ±3.09	+1.74	0.12
TG (mg/dL)	108.28 ± 4.54	117.75 ± 5.67	+9.47	**0.01**
TSH (μUI/mL)	3.65 ± 0.37	3.93 ± 0.41	+0.28	0.33
FT3 (pg/mL)	3.24 ± 0.04	3.39 ± 0.04	+0.15	0.06
FT4 (ng/dL)	1.09 ± 0.02	1.13 ± 0.02	+0.04	0.32
Vitamin D (ng/mL)	28.91 ± 2.99	29.52 ± 0.97	+0.61	0.24
Vitamin B12 (pg/mL)	400.25 ± 16.10	397.85 ± 16.16	−2.40	0.77
Insulin (μUI/mL)	10.55 ± 0.66	11.72 ± 1.23	+1.16	0.63
Intact Parathormone (pg/mL)	66.30 ± 4.19	54.37 ± 2.20	−11.92	**0.0004**

StepFit-18K was standardized on 6000 steps per 60′ of walking for three days per week for three months. * Data reported as mean and SEM for continuous variables. ^^^ Wilcoxon matched-pairs signed-rank test. Abbreviations—HbA1c: glycosylated hemoglobin; eGFR: estimated glomerular filtration rate; DB: direct bilirubin; TB: total bilirubin; AST: aspartate transaminase; ALT: alanine transaminase; GGT: gamma glutamyl transpeptidase; HDL: high-density lipoprotein; LDL: low-density lipoprotein; TC: triglyceride; TSH: thyrotropin; FT3: triiodothyronine; FT4: tetraiodothyronine.

**Table 3:** Variation in IBS-SSS parameters after the StepFit-18K intervention.

Parameters *	StepFit-18K		Δ_After-Before_	*p* ^^^
	Before	After		
Abdominal pain intensity	17.45 ± 2.62	8.40 ± 1.66	−9.06	**<0.0001**
Abdominal pain frequency	13.07 ± 2.26	5.80 ± 1.46	−7.27	**<0.0001**
Abdominal distension severity	29.13 ± 2.58	17.73 ± 1.93	−11.40	**<0.0001**
Dissatisfaction with bowel habits	31.02 ± 2.93	22.35 ± 2.18	−8.66	**0.004**
Interference with life in general	27.63 ± 2.70	20.17 ± 2.36	−7.46	**0.006**
Total Score	118.30 ± 9.55	74.46 ± 7.28	−43.84	**<0.0001**
Bristol Scale (%)				**0.02** ^Ψ^
Constipation	29 (27.36)	15 (14.15)	−13.21%	
Normal	75 (70.75)	88 (83.02)	+12.27%	
Diarrhea	2 (1.89)	3 (2.83)	+0.94%	
Mucus in Feces (Yes) (%)	10 (9.43)	7 (6.73)	−2.70%	0.18 ^Ψ^
Blood in Feces (Yes) (%)	3 (2.83)	2 (1.92)	−0.91%	0.32 ^Ψ^
Fecal urgency (Yes) (%)	36 (33.96)	18 (17.31)	−16.65%	**0.0002** ^Ψ^
Straining defecation (Yes) (%)	30 (28.30)	21 (20.19)	−8.11%	0.07 ^Ψ^
Sensation incomplete evacuation (Yes) (%)	63 (59.43)	42 (40.78)	−18.65%	**0.0003** ^Ψ^

StepFit-18K was standardized on 6000 steps per 60′ of walking for three days per week for three months. * Data reported as mean and SEM for continuous variables, and frequency and percentage (%) for categorical variables. ^ Wilcoxon matched-pairs signed-rank test; ^Ψ^ McNemar or McNemar–Bowker test where necessary.

## Data Availability

The datasets used and/or analyzed during the current study are available from the corresponding author upon reasonable request.
